# Prehospital invasive arterial blood pressure monitoring in critically ill patients attended by a UK helicopter emergency medical service– a retrospective observational review of practice

**DOI:** 10.1186/s13049-024-01193-2

**Published:** 2024-03-12

**Authors:** Emma D. Butterfield, James Price, Marco Bonsano, Kate Lachowycz, Zachary Starr, Christopher Edmunds, Jon Barratt, Rob Major, Paul Rees, Ed B. G. Barnard

**Affiliations:** 1 Department of Research, Audit, Innovation, and Development (RAID), East Anglian Air Ambulance, Norwich, UK; 2 Emergency Department, Cambridge University Hospitals NHS Foundation Trust, Cambridge, UK; 3 EuReCa, PACE Section, Department of Medicine, Cambridge University, Cambridge, UK; 4 Academic Department of Military Emergency Medicine, Royal Centre for Defence Medicine (Research & Clinical Innovation), Birmingham, UK; 5 Emergency Department, University Hospitals of North Midlands NHS Trust, Stoke-on-Trent, UK; 6 Barts Heart Centre, London, UK; 7 The Blizard Institute, Queen Mary University of London, London, UK; 8 Academic Department of Military Medicine, Royal Centre for Defence Medicine (Research & Clinical Innovation), Birmingham, UK; 9 Emergency and Critical Care Departments, Peterborough City Hospital, North West Anglia Foundation Trust, Peterborough, UK; 10 University of East Anglia, Norwich, UK

**Keywords:** Vascular Access, Blood pressure, Prehospital, Intra-arterial blood pressure, Air Ambulance, Helicopter Emergency Medical services

## Abstract

**Background:**

Accurate haemodynamic monitoring in the prehospital setting is essential. Non-invasive blood pressure measurement is susceptible to vibration and motion artefact, especially at extremes of hypotension and hypertension: invasive arterial blood pressure (IABP) monitoring is a potential solution. This study describes the largest series to date of cases of IABP monitoring being initiated prehospital.

**Methods:**

This retrospective observational study was conducted at East Anglian Air Ambulance (EAAA), a UK helicopter emergency medical service (HEMS). It included all patients attended by EAAA who underwent arterial catheterisation and initiation of IABP monitoring between 1st February 2015 and 20th April 2023. The following data were retrieved for all patients: sex; age; aetiology (medical cardiac arrest, other medical emergency, trauma); site of arterial cannulation; operator role (doctor/paramedic); time of insertion and, where applicable, times of pre-hospital emergency anaesthesia, and return of spontaneous circulation following cardiac arrest. Descriptive analyses were performed to characterise the sample.

**Results:**

13,556 patients were attended: IABP monitoring was initiated in 1083 (8.0%) cases, with a median age 59 years, of which 70.8% were male. 546 cases were of medical cardiac arrest: in 22.4% of these IABP monitoring was initiated during cardiopulmonary resuscitation. 322 were trauma cases, and the remaining 215 were medical emergencies. The patients were critically unwell: 981 required intubation, of which 789 underwent prehospital emergency anaesthesia; 609 received vasoactive medication. In 424 cases IABP monitoring was instituted en route to hospital.

**Conclusion:**

This study describes over 1000 cases of prehospital arterial catheterisation and IABP monitoring in a UK HEMS system and has demonstrated feasibility at scale. The high-fidelity of invasive arterial blood pressure monitoring with the additional benefit of arterial blood gas analysis presents an attractive translation of in-hospital critical care to the prehospital setting.

**Supplementary Information:**

The online version contains supplementary material available at 10.1186/s13049-024-01193-2.

## Introduction

Accurate haemodynamic monitoring of critically unwell and injured patients is essential to identify adverse physiology and allow titration of interventions such as volume replacement, inotropes, and vasopressors. The standard for blood pressure monitoring in the prehospital setting is non-invasive blood pressure (NIBP) measurement, which is susceptible to vibration and motion artefact, compromising its accuracy [1]. These inaccuracies are most prevalent in hypotensive and hypertensive patients– pathophysiological states frequently observed in Helicopter Emergency Medical Service (HEMS) patients [1, 2].

Invasive arterial blood pressure (IABP) monitoring is the standard of care for in-hospital critical care and presents a potential solution to the limitations observed with NIBP in the prehospital setting, where comparable levels of monitoring accuracy should ideally be obtained [3]. It is currently unclear if prehospital IABP monitoring is feasible at scale, but the combination of higher-fidelity data and the ability to continue this level of monitoring into the early in-hospital phase of care may present the optimal solution [1, 4].

Previous work describes prehospital arterial catheterisation for IABP monitoring in small cohorts of patients, predominantly at physician discretion [4–6]. In a recent survey of UK HEMS, 78% of respondents stated that IABP monitoring offers benefits to patient management [7]. However, there are no descriptions of large numbers of prehospital IABP interventions, and none from the UK. East Anglian Air Ambulance (EAAA) HEMS is one of the largest providers of prehospital critical care in the UK and has been performing arterial catheterisation and IABP monitoring for the past ten years. The aim of this study was to describe a large series of patients in whom prehospital IABP monitoring was successfully established within a UK HEMS operation.

## Methods

### Setting

EAAA provides prehospital critical care in support of the statutory emergency medical service in the East of England (East of England Ambulance Service NHS Trust). EAAA operates from two bases (Cambridge and Norwich), deploying a prehospital critical care team comprised of a physician and critical care paramedic, in either an Airbus H145 helicopter or rapid response vehicle, depending on patient location, weather constraints, and time of day [8]. EAAA predominantly responds to pre-hospital emergencies, but undertakes a small number of inter-hospital transfers.

As previously described, [8] EAAA physicians are predominantly from an emergency medicine or anaesthesia background with at least six years of post-graduate clinical experience, and competent in arterial catheterisation. Critical Care Paramedics (CCP) receive training in peripheral arterial catheterisation and point-of-care-ultrasound (POCUS), including vascular access, and then practice under physician supervision until achieving independent sign-off.

Arterial catheterisation has been performed by EAAA clinicians since 2014. A 20G arterial cannula (BD Arterial Cannula with Flow Switch, Sandy, UT, USA) and an arterial pressure transducer (Edwards Truwave 3 cc, Edwards Lifesciences, Germany) are currently used for peripheral arterial cannulation. Additionally, femoral arterial cannulation using a 5Fr sheath introducer (MERIT Prelude, MERIT Medical, South Jordan, UT, USA) has been added to the list of standard procedures since June 2021. Ultrasound guidance is mandated for femoral catheterisation and is available for radial artery catheterisation at the discretion of the operating clinician (Butterfly iQ, Butterfly Net Inc, MA, US).

Ultrasound-guided femoral arterial cannulation is formally trained as part of the local Specialist Percutaneous Emergency Aortic Resuscitation (SPEAR) programme utilising a Seldinger technique [9, 10].

### Inclusion criteria

This retrospective observational study included all patients attended by EAAA who underwent arterial catheterisation and initiation of IABP monitoring between 1st February 2015 (initiation of the electronic medical record) and 20th April 2023 (last data available at data extraction). Patients without a single systolic IABP > 20mmHg were excluded.

To ensure spurious and artefactual readings were not included in the analysis, cases meeting the following criteria were manually reviewed: <10 min of data recorded; all IABP measurements < 50mmHg; or all systolic IABP measurements were < 90mmHg. During manual review, the clinical notes and the IABP data were compared to determine whether the results were artefactual (Table S1). Cases of inter-hospital transfers were excluded from analysis as IABP monitoring is frequently initiated in-hospital prior to transfer.

### Data collection

The following data were retrieved: sex; age in years; aetiology (medical cardiac arrest, other medical emergency, trauma); anatomical site of arterial cannulation; operator role (physician/CCP); mission result (conveyed by air, conveyed by road, or died at scene). The time of insertion and, where applicable, time of pre-hospital emergency anaesthesia (PHEA), and return of spontaneous circulation (ROSC) following cardiac arrest were also retrieved.

To characterise interventions associated with IABP measurement, data were collected on the following: arterial blood gas analysis (CG8 + cartridge, iSTAT 1, Abbot Point of Care Inc, Abbot Park, IL, USA), intubation, PHEA, administration of blood products or intravenous crystalloid fluids, and vasoactive drugs (metaraminol, ephedrine, adrenaline).

The EAAA clinical governance database (RLDatix, Richmond, UK) was cross-referenced to identify any serious untoward incidents associated with arterial catheterisation.

IABP measurements were routinely downloaded from the prehospital monitor (ZOLL X Series Monitor/Defibrillator, ZOLL Medical Corporation of Asahi Kasei Corp., Tokyo) to the electronic medical record (HEMSbase, Medic One Systems Ltd, UK). Anonymised data were extracted from HEMSbase and stored in a secure data environment in Excel (Microsoft® Excel® for Microsoft 365, v2309), with data management and statistical analyses performed by a trained statistician using the R statistical programming language (R Core Team [2018]; R: A language and environment for statistical computing [R Foundation for Statistical Computing, Vienna, Austria]). Characteristics of the sample were described as number (percentage) for categorical variables and mean (± standard deviation (SD)) or median (interquartile range (IQR)) for continuous variables as appropriate.

### Ethical review

This study met the UK Health Research Authority definition of service evaluation, and was registered with the EAAA Department of Research, Audit, Innovation, & Development (REF: EAAA 2023/01). The STROBE (Strengthening the Reporting of Observational studies in Epidemiology) reporting guideline was followed [11].

## Results

**Fig. 1:**
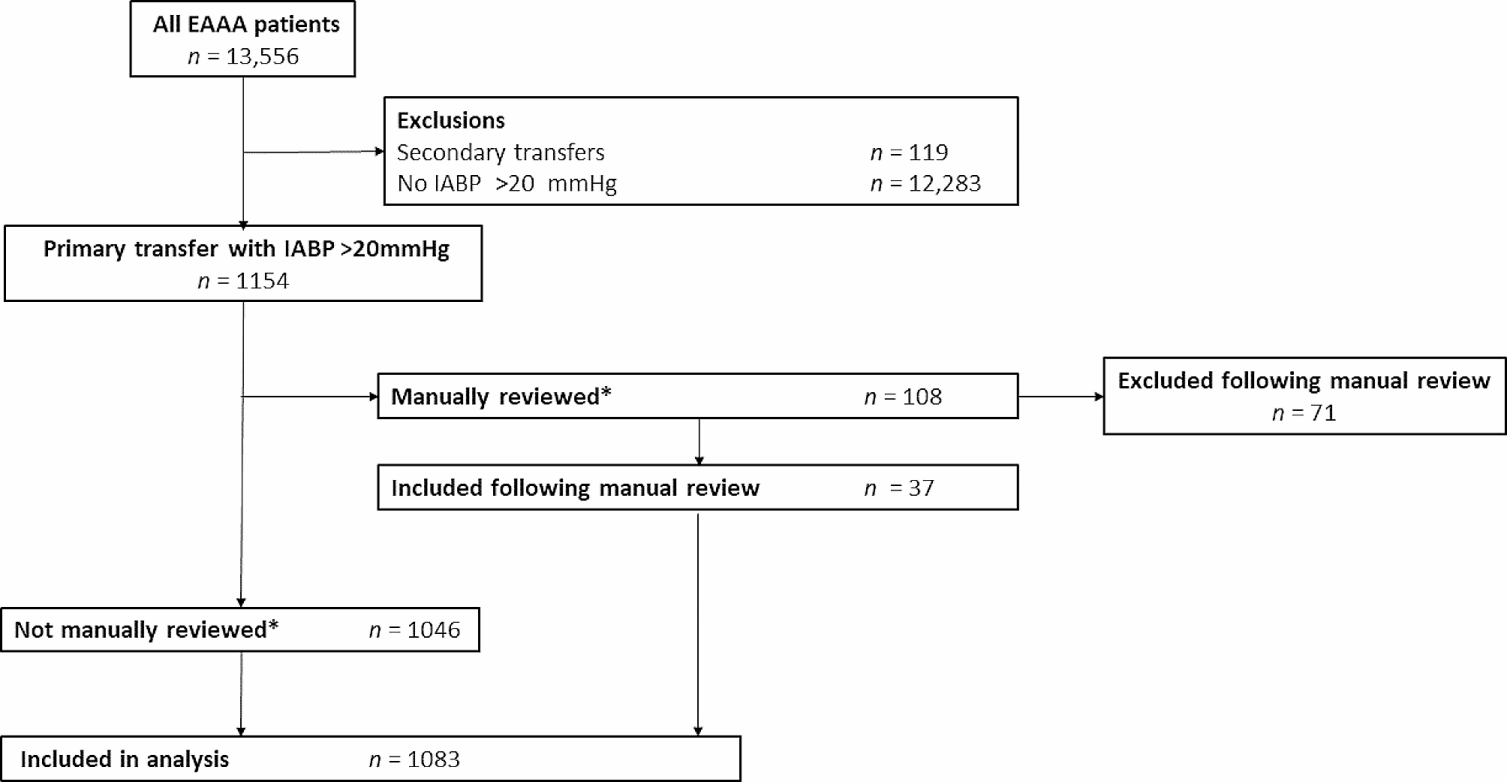
Study flow diagram of patients attended by EAAA who underwent arterial catheterisation and initiation of IABP monitoring; 01/02/2015-20/04/2023. EAAA = East Anglian Air Ambulance; IABP = intra-arterial blood pressure. *Cases were manually reviewed if there were less than 10 min of IABP measurements recorded, or all systolic IABP measurements were less than 90mmHg, or all mean arterial pressures on IABP were less than 50mmHg

During the study period 13,556 patients were attended by EAAA. IABP monitoring was successfully initiated in 1083 (8.0%) of cases, which were included in the analysis; per protocol, Fig. 1.

### Cohort description

The patients in the analysis had a median age of 59 years, and were 70.8% male. IABP monitoring was predominantly initiated in medical cardiac arrest patients (50.4%). In 22.4% of cardiac arrest patients IABP monitoring was established during cardiopulmonary resuscitation (CPR), with the remainder occurring after ROSC.

*N* = 789 patients who underwent PHEA had IABP monitoring established (22.1% of all PHEA cases during the study period); in 166 (21.0%) IABP monitoring was initiated prior to induction of anaesthesia. The most prevalent anatomical site for catheterisation was the radial artery (*n* = 869, 80.2%), and *n* = 101 femoral catheterisations were performed, Table 1.

**Table 1:** Characteristics of patients with prehospital arterial line insertion and invasive arterial blood pressure monitoring (2014–2023), *n* = 1083

Male, n (%)	767 (70.8%)
Female, n (%)	314 (29.0%)
Unknown/other	2
Age in years, median [IQR]	59.0 [46.0–70.0]
Patient aetiology	
Medical cardiac arrest, n (%)	546 (50.4%)
Trauma, n (%)	322 (29.7%)
Other Medical, n (%)	215 (19.9%)
GCS score at primary survey	
3/15, n (%)	592 (54.7%)
4–8/15, n (%)	285 (26.3%)
9–15/15, n (%)	171 (15.8%)
Not recorded, n (%)	35 (3.2%)
Anatomic site of arterial catheterisation	
Radial artery, n (%)	869 (80.2%)
Femoral artery, n (%)	101 (9.3%)
Brachial artery, n (%)	67 (6.2%)
Other/undocumented, n (%)	46 (4.3%)
Associated interventions	
ABG analysis, n (%)	591 (54.6%)
Intubation, n (%)	981 (90.6%)
Prehospital emergency anaesthesia, n (%)	789 (72.6%)
Vasoactive drugs, n (%)	609 (56.2%)
Fluid administration, n (%)	156 (14.4%)
Blood products (since March 2018*), n (%)	47 (5.6%*)

GCS = Glasgow Coma Scale; ABG = arterial blood gas; *Blood products have been available routinely, outside of a trial context, since March 2018; during the study period 276 patients received a blood product transfusion

### Professional group performing arterial catheterisation

In *n* = 893 (82.5%) of cases the professional group performing arterial catheterisation was documented in the clinical notes; physicians performed the majority of cannulations (*n* = 704, 78.8%); CCPs performed the remainder (*n* = 189).

### Timing of invasive arterial blood pressure monitoring

The median time interval from HEMS team arrival with patient to initiation of IABP monitoring was 27 (IQR: 15–42) minutes. IABP monitoring was often initiated during transport en route to hospital (*n* = 424, 39.2%): *n* = 196 (35.9%) in medical cardiac arrest patients; *n* = 151 (46.9%) in trauma patients, *n* = 77 (35.8%) in other medical patients (Table S2).

### Changes in arterial catheterisation over time

We observed a substantial increase in the proportion of patients receiving IABP monitoring through the study period: from 3.1% in 2015, to 14.4% in 2022– more than a four-fold increase.

**Fig. 2:**
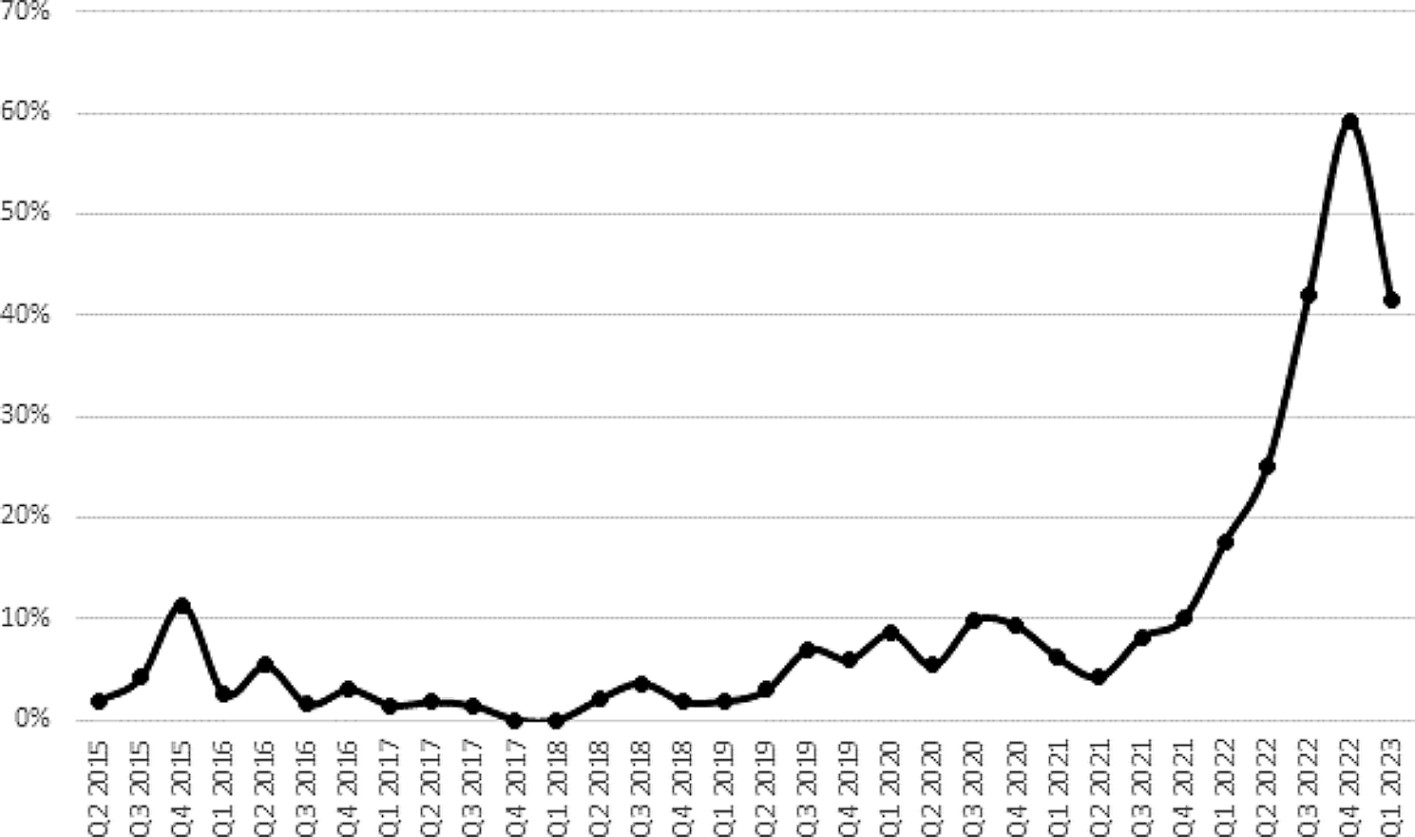
The proportion of patients that received prehospital emergency anaesthesia who had initiation of invasive arterial blood pressure monitoring prior to induction of anaesthesia (2015–2023)

This change was driven by an increase in the use of IABP monitoring in the cohort of patients that received PHEA (who comprised 72.6% of all patients who received IABP monitoring). Within the group of patients receiving PHEA, towards the end of the study period, there was an increase in the proportion in whom IABP monitoring was initiated *prior* to PHEA, representing more than 40% of PHEA patients from mid-2022 onwards (Fig. 2).

### Arterial catheterisation complications

No immediate complications were reported during the study period. In hospital, one patient developed acute limb ischemia requiring forearm fasciotomy and thrombectomy following prehospital catheterisation of the brachial artery. An independent in-hospital formal review of the case concluded that the ischemia was either due to profound systemic hypotension or arterial dissection caused by catheterisation.

## Discussion

This study reports more than one thousand cases of arterial catheterisation and initiation of IABP monitoring in the prehospital phase of care for seriously unwell and injured patients. The most prevalent patient aetiology was medical cardiac arrest, and the majority of arterial lines were inserted peripherally in the radial artery. Almost all of the patients were intubated, and over half also benefited from arterial blood gas monitoring. In this system, one in five arterial lines were inserted by non-physicians, approximately 40% were inserted and transduced during transport, and there was only one reported potential arterial catheter-associated complication in ten years of practice.

A quarter of all cases attended during the study period (*n* = 3453) had experienced a medical cardiac arrest, and often required advanced airway and post-ROSC circulatory support (including PHEA and administration of titrated inotropes). In this study, more than half of all arterial catheterisations were performed in patients presenting with OHCA (catheterised either during resuscitation or following ROSC). The use of IABP monitoring in these patients has been recommended in clinical guidelines [12], but this has been perceived as challenging to deliver in the prehospital environment. The benefits of IABP monitoring intra-arrest include the ability to measure the efficacy of chest compressions, and to estimate coronary perfusion pressure (CPP). Previous studies have demonstrated the positive association between a CPP > 15mmHg and ROSC [13, 14], therefore when IABP monitoring has been established, individualized treatment can occur and the response to therapy can be measured beat-to-beat in real-time. Following ROSC, IABP monitoring allows for immediate detection of re-arrest and episodes of critical hypotension and can be used to accurately titrate vasopressors and inotropic medications.

This study demonstrates that IABP monitoring can be delivered in this cohort and should therefore be considered by all HEMS providing critical care to medical cardiac arrest patients.

Blunt head injury with associated loss of consciousness is a prevalent indication for UK HEMS deployment, due to the potential need for neuroprotective measures, including PHEA [15]. Concomitant head injury was seen in 48% of critically injured, hypotensive trauma patients attended to by UK HEMS [16]. Hypotension and hypertension in patients with traumatic brain injury is associated with increased mortality [17]. Approximately one in five patients who undergo PHEA have post-induction hypotension within the first ten minutes of induction [2]. Following the publication of this study, EAAA updated its PHEA standard operating procedure in July 2022, including a recommendation that clinicians consider IABP monitoring when performing PHEA, ideally prior to anaesthesia, in order to rapidly detect and mitigate haemodynamic compromise. Although there was no change in the total number of PHEA per quarter (median 56, IQR: 49–63), the number of PHEA patients who had IABP monitoring increased from 2021 onwards. Since late 2022, more than 40% of patients receiving a PHEA had IABP monitoring initiated prior to induction of anaesthesia, demonstrating this level of monitoring is feasible at scale in the prehospital environment for the most critically injured patients and should be considered by HEMS providing PHEA. Since publication of this new operating procedure, we have also reported that > 10% of PHEA patients experience post-induction critical hypertension [18], further emphasising the importance of high-fidelity blood pressure monitoring in this vulnerable group. However, in patients with critical airway compromise, or other time-critical pathology, PHEA may take priority over arterial catheterisation. Pre-PHEA IABP monitoring remains at clinician discretion, reflected by the fluctuations in its utilisation since 2022.

Scene times were not reported, firstly because the group in which IABP monitoring was not successfully instituted contains patients in whom arterial catheterisation was attempted and failed, and is therefore not an effective comparator. Secondly, recent evidence has shown that some patients benefit from a longer scene time, to allow the optimal delivery of prehospital critical care, which may include initiation of IABP monitoring [19]: further research is necessary to determine which patient groups benefit from particular interventions.

Most arterial catheters were placed in the radial artery, where collateral flow through the ulnar artery reduces the risk of distal ischaemia that might be caused by radial arterial occlusion, spasm, or thrombosis. Whilst no immediate complications of arterial cannulation were reported during the study period, further work is required to explore any longer-term complications and the longevity of arterial catheters inserted prehospital. Estimating the risk of arterial dissection or thrombosis following arterial line placement is challenging: a large retrospective study of complications following peri-operative arterial catheterisation in hospital [20] reported a complication rate per 10,000 cannulations of 2.7 (95% CI 1.5–4.4) for radial cannulation, and 12.3 (95% CI 1.5–44.4) for brachial catheterisation. There, brachial catheterisation was performed in only 2.6% of patients, with the relative paucity of data making estimation of the complication rate less accurate. However, a much higher complication rate is reported in critically unwell patients in the intensive care unit (ICU)– a study of 4932 arterial catheterisations reported distal vascular insufficiency in 3.8% of cases [21]. A retrospective observation study of 53 ICU patients in whom the brachial artery was catheterised reported 6 cases of distal ischaemia and one of distal thrombosis [22], in a group of patients with 57% mortality. These data suggest that the complication rate of brachial cannulation may be associated with the severity of the patient’s physiological derangement. Our study is too small to determine with the rate of complications of pre-hospital arterial cannulation, but our one reported complication following 67 catheterisations of the brachial artery, in critically unwell patients, is favourably comparable with the complication rate reported in the ICU population. Catheterisation of the radial artery is preferred to the brachial, due to the presence of collateral distal flow via the ulnar artery: typically, the brachial artery is only used when radial and/or femoral access is not feasible; brachial artery catheterisation is only performed where the clinician believes the benefit to the patient outweighs the known risks.

### Limitations

Whilst this is the largest study describing prehospital arterial catheterisation and IABP monitoring, the data are retrospective from a single centre and are limited by the challenging nature of prehospital data collection. This study is not able to report overall catheterisation success rate or detail the mechanics of arterial catheterisation (number of attempts, utilisation of ultrasound, professional group success rate). This precludes analysis of factors contributing to failure, or delays to insertion. Similarly, the group of patients in whom IABP was not measured includes an unknown number of patients in whom arterial catheterization may have been attempted but ultimately abandoned, so cannot be used as a comparator group.

## Conclusion

This study describes over 1000 cases of prehospital arterial catheterisation and invasive blood pressure monitoring in a UK HEMS system and has demonstrated feasibility at scale. The high-fidelity of invasive arterial blood pressure monitoring with the additional benefit of arterial blood gas analysis presents an attractive translation of in-hospital critical care to the prehospital setting.

### Electronic supplementary material

Below is the link to the electronic supplementary material.

Supplementary Material 1

Supplementary Material 2

Supplementary Material 3

## Data Availability

The datasets used and analysed in this study are available from the corresponding author on reasonable request.

## References

[1] McMahon N, Hogg LA, Corfield AR, Exton AD (2012). Comparison of non-invasive and invasive blood pressure in aeromedical care. Anaesthesia.

[2] Price J, Moncur L, Lachowycz K (2023). Predictors of post-intubation hypotension in trauma patients following prehospital emergency anaesthesia: a multi-centre observational study. Scand J Trauma Resusc Emerg Med.

[3] Lockey DJ, Crewdson K, Lossius HM (2014). Prehospital anaesthesia: the same but different. Br J Anaesth.

[4] Sende J, Jabre P, Leroux B, Penet C, Lecarpentier E, Khalid M, Margenet A, Marty J, Combes X (2009). Invasive arterial blood pressure monitoring in an out-of-hospital setting: an observational study. Emerg Med J.

[5] Wildner G, Pauker N, Archan S, Gemes G, Rigaud M, Pocivalnik M, Prause G (2011). Arterial line in prehospital emergency settings - a feasibility study in four physician-staffed emergency medical systems. Resuscitation.

[6] Fok PT, Teubner D, Purdell-Lewis J, Pearce A (2019). Predictors of Prehospital On-Scene Time in an Australian emergency Retrieval Service. Prehosp Disaster Med.

[7] Morton S, Avery P, Payne J, OMeara M (2022). Arterial blood gases and arterial lines in the Prehospital setting: a Systematic Literature Review and survey of current United Kingdom Helicopter Emergency Medical services. Air Med J.

[8] Price J, Lachowycz K, Steel A, Moncur L, Major R, Barnard EBG (2022). Intubation success in prehospital emergency anaesthesia: a retrospective observational analysis of the Inter-changeable Operator Model (ICOM). Scand J Trauma Resusc Emerg Med.

[9] Chana M, Perkins Z, Lendrum R, Sadek S (2021). A practical Approach to Introducing Pre-hospital resuscitative endovascular balloon occlusion of the aorta (REBOA), the problems encountered and lessons learned. J Endovascular Resusc Trauma Manage.

[10] Brede JR, Lafrenz T, Krüger AJ et al. Resuscitative endovascular balloon occlusion of the aorta (REBOA) in non-traumatic out-of-hospital cardiac arrest: evaluation of an educational programme BMJ Open 2019. 10.1136/bmjopen-2018-027980. 10.1136/bmjopen-2018-027980 PMC6528011 31076474

[11] von Elm E, Altman DG, Egger M, Pocock SJ, Gøtzsche PC, Vandenbroucke JP, STROBE Initiative (2007). The strengthening the reporting of Observational studies in Epidemiology (STROBE) statement: guidelines for reporting observational studies. PLoS Med.

[12] Panchal AR, Bartos JA, Cabañas JG, Donnino MW, Drennan IR, Hirsch KG, Kudenchuk PJ, Kurz MC, Lavonas EJ, Morley PT, O’Neil BJ, Peberdy MA, Rittenberger JC, Rodriguez AJ, Sawyer KN, Berg KM (2020). Adult basic and Advanced Life Support Writing Group. Part 3: adult basic and advanced life support: 2020 American Heart Association Guidelines for Cardiopulmonary Resuscitation and Emergency Cardiovascular Care. Circulation.

[13] Paradis NA, Martin GB, Rivers EP (1990). Coronary perfusion pressure and the return of spontaneous circulation in human cardiopulmonary resuscitation. JAMA.

[14] Rivers EP, Lozon J, Enriquez E, Havstad SV, Martin GB, Lewandowski CA, Goetting MG, Rosenberg JA, Paradis NA, Nowak RM (1993). Simultaneous radial, femoral, and aortic arterial pressures during human cardiopulmonary resuscitation. Crit Care Med.

[15] Carney N, Totten AM, O’Reilly C, Ullman JS, Hawryluk GW, Bell MJ, Bratton SL, Chesnut R, Harris OA, Kissoon N, Rubiano AM, Shutter L, Tasker RC, Vavilala MS, Wilberger J, Wright DW, Ghajar J (2017). Guidelines for the management of severe traumatic brain Injury. Fourth Ed Neurosurg.

[16] Crombie N, Doughty HA, Bishop JRB, Desai A, Dixon EF, Hancox JM, Herbert MJ, Leech C, Lewis SJ, Nash MR, Naumann DN, Slinn G, Smith H, Smith IM, Wale RK, Wilson A, Ives N, Perkins GD (2022). RePHILL collaborative group. Resuscitation with blood products in patients with trauma-related haemorrhagic shock receiving prehospital care (RePHILL): a multicentre, open-label, randomised, controlled, phase 3 trial. Lancet Haematol.

[17] Spaite DW, Hu C, Bobrow BJ, Chikani V, Barnhart B, Gaither JB, Denninghoff KR, Adelson PD, Keim SM, Viscusi C, Mullins T, Rice AD, Sherrill D (2017). Association of Out-of-Hospital Hypotension Depth and duration with traumatic brain Injury Mortality. Ann Emerg Med.

[18] Sagi L, Price J, Lachowycz K, Starr Z, Major R et al. Critical hypertension in trauma patients following prehospital emergency anaesthesia: a multi-centre retrospective observational study. Scand J Trauma Resus Emerg Med. In press. 10.1186/s13049-023-01167-w. 10.1186/s13049-023-01167-w PMC10731700 38124103

[19] Knapp J, Doppmann P, Huber M (2023). Pre-hospital endotracheal intubation in severe traumatic brain injury: ventilation targets and mortality—a retrospective analysis of 308 patients. Scand J Trauma Resusc Emerg Med.

[20] Nuttall G, Burckhardt J, Hadley A, Kane S, Kor D, Marienau MS, Schroeder DR, Handlogten K, Wilson G, Oliver WC (2016). Surgical and Patient Risk factors for severe arterial line complications in adults. Anesthesiology.

[21] Frezza EE, Mezghebe H (1998). Indications and complications of arterial catheter use in surgical or medical intensive care units: analysis of 4932 patients. Am Surg.

[22] Schellenberg M, Hawley L, Biswas S, Clark DH, Cobb JP (2020). Complications following brachial arterial catheterization in the Surgical Intensive Care Unit. Am Surg.

