# Supplementary material for: Prehospital invasive arterial blood pressure monitoring in critically ill patients attended by a UK helicopter emergency medical service– a retrospective observational review of practice
Source: Scand J Trauma Resusc Emerg Med. 2024 Mar 12;32:20. doi: 10.1186/s13049-024-01193-2 (PMC10935774; doi:10.1186/s13049-024-01193-2)
Supplement: Supplementary file 1 — Supplementary Material 1 [file 13049_2024_1193_MOESM1_ESM.docx]

**Table S1: Inclusion and exclusion of cases following manual review.**

| Criteria for manual review | Included following manual review | Excluded following manual review | Total |
| --- | --- | --- | --- |
| < 10 minutes IABP data | 8 | 11 | 19 |
| All mean arterial pressures on IABP measurement <50mmHg | 15 | 47 | 62 |
| All systolic IABP measurements <90mmHg | 23 | 41 | 64 |

There was overlap between the categories, with some cases falling into more than one category: in total 108 cases were manually reviewed: 71 were excluded and 37 included.
