# Supplementary material for: Prehospital invasive arterial blood pressure monitoring in critically ill patients attended by a UK helicopter emergency medical service– a retrospective observational review of practice
Source: Scand J Trauma Resusc Emerg Med. 2024 Mar 12;32:20. doi: 10.1186/s13049-024-01193-2 (PMC10935774; doi:10.1186/s13049-024-01193-2)
Supplement: Supplementary file 2 — Supplementary Material 2 [file 13049_2024_1193_MOESM2_ESM.docx]

**Table S2: IABP monitoring and timing of initiation by patient group**

|  | **All** | **Medical** | **Trauma** | **OHCA** |
| --- | --- | --- | --- | --- |
| IABP monitoring, n  (% of all patients) | 1083  (8.0%) | 215  (1.6%) | 322  (2.4%) | 546  (4.0%) |
| Time from arrival to 1^st^ IABP (mins, median (IQR) ) | 27 (15 – 42) | 27 (15 – 42) | 32 (16 – 51) | 23 (13 – 37) |
| Timing of 1^st^ IABP: |  |  |  |  |
| At scene, n (%) | 659 (60.8%) | 138 (64.2%) | 171 (53.1%) | 350 (64.1%) |
| After leaving scene/in transit, n (%) | 424 (39.2%) | 77 (35.8%) | 151 (46.9%) | 196 (35.9%) |
