# Supplementary material for: Prehospital invasive arterial blood pressure monitoring in critically ill patients attended by a UK helicopter emergency medical service– a retrospective observational review of practice
Source: Scand J Trauma Resusc Emerg Med. 2024 Mar 12;32:20. doi: 10.1186/s13049-024-01193-2 (PMC10935774; doi:10.1186/s13049-024-01193-2)
Supplement: Supplementary file 3 — Supplementary Material 3 [file 13049_2024_1193_MOESM3_ESM.docx]

Reviewer reports:

Reviewer #1: The authors have responded constructively to reviewer suggestions and the revised submission has benefitted substantively from the ammendments.
I have no further comments or suggestions other that accept.

Thank you for your comments which have contributed to the betterment of the manuscript.

Reviewer #2: Thank you for considering both reviewers' comments, which have improved the manuscript!

Thank you for your considered critique, which has improved the manuscript.

I still have two comments regarding complications and on-scene time:

The case review concluded that the catheterisation may have been the cause of the limb ischemia. Thus, the reader may find the statement in the abstract and conclusion of no clear signal of catheter-related complication as bold. The manuscript would benefit from discussing the complication rate you experienced compared to the in-hospital setting and concerning the insertion site. (Based on your opinion, do you still recommend a brachial artery line?) As commented by the other reviewer, be frank about the risk.

In view of this more nuanced appraisal of risk now included in the manuscript, the blanket statement of “no clear signal of catheter associated complications” has now been removed from the abstract and conclusion. The discussion now references in-hospital complication rates, and the conclusion now refers to the complication rate being comparable to in-hospital practice.

You have added a section regarding on-scene time in the discussion, which is good. However, I would question the "paradigm shift" statement in the last sentence. It is correct that "shorter is not always better", but more research is needed regarding patient groups and interventions. The last sentence is, in my opinion, not required.

The last sentence has been removed, and replaced with a more concise statement that “further research is necessary to determine which patient groups benefit from particular interventions”, which we believe encapsulates the key message about the potential benefits and uncertainties around longer scene times.

Otherwise, a well written manuscript!

Thank you for your helpful critique. An extended discussion of the evidence around the risks of arterial catheterisation has now been included, referencing both the rates of complications associated with perioperative arterial catheterisation, and the rates of complications reported in critically unwell ICU patients (line 255 onwards)
